# Pharmacogenetics Informed Decision Making in Adolescent Psychiatric Treatment: A Clinical Case Report

**DOI:** 10.3390/ijms16034416

**Published:** 2015-02-20

**Authors:** Teri Smith, Susan Sharp, Ann M. Manzardo, Merlin G. Butler

**Affiliations:** Department of Psychiatry and Behavioral Sciences, University of Kansas Medical Center, 3901 Rainbow Boulevard, Kansas City, Kansas 66160, USA; E-Mails: ssharp@kumc.edu (S.S.); amanzardo@kumc.edu (A.M.); mbutler4@kumc.edu (M.G.B.)

**Keywords:** pharmacogenetics, cytochrome p450 enzymes, psychotropic medications, psychiatry, behavior, autism, clinical case

## Abstract

Advances made in genetic testing and tools applied to pharmacogenetics are increasingly being used to inform clinicians in fields such as oncology, hematology, diabetes (endocrinology), cardiology and expanding into psychiatry by examining the influences of genetics on drug efficacy and metabolism. We present a clinical case example of an adolescent male with anxiety, attention deficit hyperactivity disorder (ADHD) and autism spectrum disorder who did not tolerate numerous medications and dosages over several years in attempts to manage his symptoms. Pharmacogenetics testing was performed and DNA results on this individual elucidated the potential pitfalls in medication use because of specific pharmacodynamic and pharmacokinetic differences specifically involving polymorphisms of genes in the cytochrome p450 enzyme system. Future studies and reports are needed to further illustrate and determine the type of individualized medicine approach required to treat individuals based on their specific gene patterns. Growing evidence supports this biological approach for standard of care in psychiatry.

## 1. Introduction

Pharmacogenetics is the field of study that examines the influence of genetics on drug efficacy and tolerability often based on the cytochrome p450 enzymes involved in drug metabolism encoded by a large family of protein-coding genes [1]. Cytochrome p450 (CYP450) enzymes are present in most bodily tissues primarily positioned within the inner mitochondrial membrane or endoplasmic reticulum of cells. They are perhaps best known for their function in the metabolism of potentially toxic compounds including metabolic byproducts (e.g., bilirubin) and drugs but play an important role in the biosynthesis and metabolism of lipids, steroids, including hormones, and select vitamins [1]. PharmacoGENETICS can be discriminated from the related field of “PharmacoGENOMICS” which considers the broader influences of inheritance on gene products, expression and function outside the limited scope of the cytochrome p450 enzyme system, primarily found in the liver. Natural variation in sensitivity and action of cytochrome p450 enzymes contributes to variations in drug response and side effect profiles of direct relevance to medical management of drug therapy. For example, about one in every 15 individuals show an exaggerated response to standard doses of beta blockers, a class of medications commonly prescribed for hypertension and metabolized by the CYP450 enzyme system and when disturbed leads to side effects [2,3].

CYP450 enzymes are encoded by genes with the wild-type allele occurring in most individuals; however, an extensive or normal metabolizer receives two copies of the wild-type allele. The presence of other allele variants usually indicates reduced or no CYP450 enzyme activity. Individuals with two copies of a variant allele of one of the genes encoding a CYP450 enzyme are considered poor metabolizers while individuals with one wild-type allele and one variant allele have significantly reduced enzymatic activity. Individuals who inherit multiple copies of the wild-type allele are generally extensive metabolizers or ultrametabolizers and degrade drugs quickly [2,3].

Despite the relatively large number and broad function of the cytochrome p450 enzyme superfamily with over 50 members, 90 percent of all drugs are metabolized by just six different enzymes (CYP1A2, CYP3A5, CYP2C19, CYP2D6, CYP3A4 and CYP3A5) which significantly limit the number of genetic targets needed for screening and enhancing clinical utility [2]. Clinical testing platforms tailored for medical specialties are now available and typically provide interpretive services for treatment and dosage recommendations based upon the genetic testing results. Interpretive insight is particularly important for clinicians and hospitals that may be unfamiliar or apprehensive about the application of novel technologies in diagnosis and treatment in changing medical practice.

Pharmacogenetics have been used successfully to optimize treatment in cardiology, diabetes, and oncology [4,5], and has been particularly helpful for psychiatric medications which account for 20% of the 121 pharmacogenetic markers currently recognized by the US Food and Drug Administration [6]. Pharmacogenetics testing typically targets single nucleotide polymorphisms (SNPs) of the top six CYP450 enzyme genes which are now utilized in many areas of medicine including psychiatry for drug selection and adjustment to improve efficacy and reduce medication adverse events [7]. Potential financial and personal costs of adverse drug events, including deaths, and effectiveness in treatment and reducing psychiatric costs may be helped by providing better informed decision making when prescribing psychotropic drugs as outlined in a recent report by Durham [8]. The American Medical Association has made similar suggestions regarding how pharmacogenetics can reduce healthcare costs by decreasing the number of adverse drug reactions and number of medications used by patients to yield more effective therapies [9]. The following detailed clinical case history and report is presented as an example of how pharmacogenetics and the practice of psychiatry can interface now and in the future. In this case, pharmacogenetics testing provided more comprehensive and informed psychiatric care decision-making and evaluation as well as therapeutic outcomes beneficial for the patient and immediate family.

## 2. Results and Discussion

### 2.1. Clinical Case Report

Our 12-year-old Caucasian male was first evaluated by his current psychiatrist in April 2010 after years of seeking care from other psychiatrists and psychotherapists during his childhood due to disruptive behaviors. His history included psychiatric hospitalization for 2 days while on fluoxetine at 10 years of age and occupational therapy for sensory issues at 12 years of age. He had received a variety of treatments from psychotherapists. His past diagnoses included Attention Deficit Hyperactivity Disorder (ADHD), combined type, since 7 years of age, Anxiety Disorder-Not Otherwise Specified (NOS), learning problems, Obsessive Compulsive Disorder, chronic bed-wetting at a younger age, Pervasive Developmental Disorder-NOS (Autism Spectrum Disorder), Sensory Integration Disorder and drug induced mania. He reported no drug allergies, food sensitivities or intolerances.

Many different psychotropic medications had been prescribed, many of which were not tolerated or helpful. At the time of his initial evaluation, he was taking oxcarbazepine (total 375 mg), lamotrigine (175 mg), fluvoxamine (75 mg) and risperidone (0.5 mg) which were of limited to no benefit in controlling his behavior. He had experienced irritability, tantrums, impulsivity, distractibility, fidgetiness and obsessive thoughts without rituals, rigidity to change, sensory sensitivities and oppositional behaviors for much of his life. He slept well and had a normal diet. Over the next two years (2010–2012), he was prescribed atomoxetine (18 mg) and stimulants (dexamfetamine (30–60 mg), dextroamphetamine (15 mg). Risperidone was replaced with aripiprazole (5–10 mg). In February 2011 his laboratory findings showed a normal hematogram, a normal comprehensive metabolic panel, normal thyroid studies and normal fasting lipid levels. A fluoxetine (5–15 mg) trial showed minimal benefit, and he did not tolerate doses higher than 15 mg. Citalopram 10 mg was added in April 2012.

He was evaluated at 14 years of age by a clinical geneticist on our team (MGB). His height was 159 cm (15%), weight was 53.8 kg (50%) and head circumference was 53.1 cm (5%). He was non-dysmorphic and no single gene disorder was identified. The family history was positive for obsessive compulsive disorder, anxiety, irritability and anger control problems, ADHD, learning problems, heart problems, bipolar disorder, stuttering and drug and alcohol addiction. A chromosomal microarray test was normal without recognized deletions or duplications in the genome. Additional laboratory studies showed a normal comprehensive metabolic panel, hematogram, cortisol (morning), C-reactive protein, insulin (fasting), somatomedin C, but his total testosterone levels were low (80 ng/dL with reference range 270–1070 ng/dL). An X-ray bone age study showed delayed bone development. A referral was then made to an endocrinologist who prescribed testosterone cypionate injections (200 mg/mL). He was also taking over the counter (OTC) supplements, fish oil and lactobacillus probiotics at this time along with OTC flaxseed oil and ω-3 fatty acids. Veema liquid vitamins were added in January 2012. All OTC supplements were given as suggested by label. There was no perceived benefit from aripiprazole (2.5 mg) and it was discontinued. He then experienced trouble sleeping with irritability and reportedly visualized shadows. Aripiprazole (2.5 mg) was again prescribed and citalopram was increased to 15 mg. Psychotherapy was suggested, but he became more hyperactive, irritable, and angry. Aripiprazole was decreased to 2 mg while citalopram was decreased to 10 mg and testosterone injections continued. In April 2013, aripiprazole was decreased to 1 mg and hypnotherapy with relaxation techniques were offered but with only small positive effects. Aripiprazole was then discontinued. He began to take OTC amino acid supplements, salmon oil, melatonin for sleep and Empower Plus Vitamin with mineral supplements as suggested by label. He was taking 10 mg of citalopram prescribed by his psychiatrist but began to experience a crawling sensation of his skin. A low dose of diphenhydramine was suggested. His mother was advised to discuss with the pharmacist the use of OTC supplements and their possible side effects.

By June 2013, he was having frequent panic attacks and tactile sensory sensitivities which often triggered explosive episodes. His citalopram was reduced to 7.5 mg and buspirone was considered as a possible next step for pharmacotherapy. By August 2013, he was off all medications other than Empower Plus Vitamins and experienced increased symptoms of inattention and anxiety about school. He was sleeping better, but became stressed prior to attending school and wearing street clothes. He saw a new psychotherapist in August 2013 at 15 years of age for constructive methods to deal with his anxiety and sensory issues. He was a high school sophomore who did well academically (A’s and B’s). He participated in cross country and swimming but experienced anger dysregulation at home. Wearing street clothes bothered him extremely causing mood irritability by the time he arrived home from school.

He reported taking the following prescription medications in the past: Fluvoxamine (25–100mg), divalproex (125 mg), clonidine (0.5 mg), guanfacine (1 mg), sertraline (25 mg), aripiprazole (2–10 mg), oxcarbazepine (375 mg), risperidone (0.5 mg), lamotrigine (175 mg), lisdexamfetamine (30–60 mg), dextroamphetamine (15 mg), citalopram (10 mg), fluoxetine (5–15 mg), atomoxetine (18 mg), quetiapine (50 mg), imipramine (25 mg), testosterone, and alprazolam (0.5 mg). He felt hopelessness at times and did not understand why this was happening to him. He was polite and had a supportive family. He was socially awkward and friendships were limited. He had problems transitioning from one activity to another. At this point, his mother was giving him OTC supplements including amino acids, salmon oil, Empower vitamins, inositol when anxious, vitamin D3, choline, and probiotics as suggested by label.

At 15 years of age, he continued to have frequent “melt-downs” in the morning and could not tolerate tactile sensory stimulation. He had anxiety attacks during which he screamed and protested loudly. He became shaky, hot, and sweaty. He did not eat well or sleep normally. On September 30, 2013 escitalopram (5 mg) was prescribed and he immediately recorded less anxiety and his sensory issues improved. He requested an increase in dosage of escitalopram immediately because of the positive effect. He then began to use coping skills more efficiently such as mindfulness, listening to music, and deep breathing as well as behavioral strategies for coping with his symptoms. At about this time, he contracted a sinus infection and was using Zyrtec and escitalopram. His anger problems became much worse and his sensory problems increased. His therapist consulted with an occupational therapist in October 2013 and he began to use exercise and joint compressions along with other behavioral strategies to decrease sensory sensitivities. The patient’s parents observed when the patient recently used OTC Nyquil Cold Medicine which contains acetaminophen, dextromethorphan and doxylamine succinate that it not only helped his cold symptoms, but his anxiety, sensory sensitivities, and other related behavioral problems lessened. When he discontinued this OTC medication, his anxiety, panic, and nail picking behaviors increased. Pharmacogenetics testing was then suggested due to his multiple episodes of behavioral problems that were not controlled over time with the use of several different medications and dosages.

Over time, escitalopram continued to help his anxiety, but sensory issues were still present. Escitalopram was increased to 10 mg on October 28, 2013 and behavioral improvement was notable with many days without melt-downs. Over the following few months he was dealing better with wearing specific clothing (e.g., long pants) and with transitions. He was better motivated to improve his grades from Bs to As and his overall school performance. He began to wear dress clothes (usually he would wear only soft, loose clothing). He wanted to socialize more by making new friends and to improve his social skills. ADHD symptoms lessened while on the increased level of escitalopram. He felt he could now take charge of his own self-soothing but could think through issues *versus* over-reacting to trivial frustrations. His social life improved and escitalopram was increased to 20 mg in June 2014. The family saw a correlation between inadequate eating and sleeping and the patient’s over-reactions. They devised a plan to remind him to eat regularly and to encourage him to obtain sufficient sleep. His sensory issues improved and he was much kinder to others. He now had a job and was able to tolerate wearing rough-textured fabric pants and a T-shirt. He excelled at work and was given a promotion.

### 2.2. Pharmacogenetics

The DNA-based pharmacogenetics Genecept assay testing (Genomind, Chalfont, PA, USA) examines polymorphisms from 10 separate genes with three genes encoding cytochrome p450 enzymes related to medication metabolism (*CYP2D6*, *CYP2C19*, *CYP3A4*) and 7 additional genes consisting of neurotransmitter receptors (*5HT2C*, *DRD2*) and transporters (*SLC6A4*), enzymes (*COMT*, *MTHFR*) and ion channel function (*CACNA1C*, *ANK3*) involved with pharmacodynamics of drug activity and interaction. The three liver cytochrome p450 enzymes selected are major metabolizers of psychiatric medications and their gene variants are determined to have clinically relevant impacts on drug interaction and metabolism in the clinical setting. The assay contains a C/C gene variation of a serotonin receptor [5-hyroxytryptamine receptor 2C (5HT2C)] that has been associated with increased weight gain with atypical antipsychotic therapy [10,11,12,13,14]. The *SLC6A4* gene codes for a presynaptic serotonin transporter protein (SERT) responsible for serotonin reuptake and targeted by most selective serotonin reuptake inhibitors (SSRIs). The *SLC6A4* gene product can produce a long (L) and short (S) length variant with different clinical significance. Possession of two S variants is associated with a poor or slow response to SSRIs or with adverse events [10,15].

The DRD2 receptor is a target of most neuroleptics which act to block signaling of the neurotransmitter dopamine. The *DRD2* variant selected (-141C Ins/Del) is a variation in the promoter region of the gene that reduces *DRD2* gene expression and responsiveness along with potential adverse events when using atypical antipsychotic medications. The *COMT* gene codes for catechol-*O*-methyl-transferase which is an enzyme responsible for the majority of dopamine metabolism. Dopamine is critical for memory, judgment, attention, and other executive functions and strongly linked to multiple neuropsychiatric disorders [16]. A valine (Val) to methionine (Met) amino acid substitution is produced by a polymorphism at codon 158 due to a nucleotide G to A transition which results in approximately 40% reduction in COMT enzymatic activity [17]. The Val/Val substitution leads to elevated enzyme activity causing increased dopamine degradation (producing low dopamine) while the Met/Met substitution produces a 3 fold reduction in enzyme activity and reduced dopamine metabolism relative to Val/Val [18]. The Val/Val substitution is associated with a hypodopaminergic state and lower executive function and implicated in susceptibility to schizophrenia, panic disorder and anorexia nervosa.

A methylenetetrahydrofolate reductase (*MTHFR*) C/T gene variation has been shown to slow the conversion of folate or folic acid to methylfolate, a precursor to serotonin, norepinephrine, and dopamine synthesis. This variant impacts monoamine and catecholamine production [19,20,21,22,23,24,25,26] and associated with depression. L-methylfolate has shown efficacy as an adjunctive therapy in individuals with Selective Serotonin Reuptake Inhibitor resistant major depression [21,22,23,24,25]. The MTHFR enzyme metabolizes homocysteine and if not broken down effectively, can build up in the blood stream and lead to health concerns related to cardiovascular disease [26].

Molecular transport and regulation of intracellular calcium levels are important in neurological development and function with pathology linked to numerous neuropsychiatric disorders including depression, schizophrenia and bipolar disorder [27]. A variant (G to A) of the α-1C subunit of the L-type voltage gated calcium channel gene (*CACNA1C*) influences the threshold for activation and duration of channel opening leading to excessive calcium influx into the cell and neuronal hypersensitivity to activating stimuli [27]. The variation predicts poor clinical response to current pharmacotherapy. Another ion channel function related gene is Ankyrin-G (ANK3) which encodes a protein located at the nodes of Ranvier and neurons responsible for the generation of action potentials. It is important for the function and maintenance of voltage dependent sodium channels. Modest evidence links a common T to G transition with schizophrenia in this specific ankyrin gene family member [27].

### 2.3. DNA-based Pharmacogenetic Results

The Genecept assay results identified in our clinical case involved three known significant variations in the 10 genes tested which impact on several pharmacologic substrates (see Table 1). Our clinical case was found to have a *5HT2C* C/C gene variation of a serotonin receptor which is associated with satiety signaling in the hypothalamus and hence, serotonin has a potent satiety signal function and thus 5HT2C antagonism can lead to increased food intake [10,11,12,13,14]. Although the weight of our clinical case was within normal limits, this finding suggested that caution be used when prescribing atypical antipsychotics such as risperidone. Our clinical case also showed a *MTHFR* C/T gene variation that suggested reduced enzymatic activity associated with a reduced conversion of folic acid to methylfolate. As methylfolate is a precursor to serotonin, norepinephrine, and dopamine, this gene variant would indicate a possible reduced production of these peptides [19,20,21,22,23,24,25,26].

Variants of *MTHFR* have been related to increased risk for depression and L-methylfolate has shown efficacy as an adjunctive therapy. It was recommended that our clinical case should take folic acid supplements or L-methylfolate to help in the conversion of homocysteine and health concerns related to cardiovascular disease [26]. Interestingly, there was a maternal family history of heart disease that may be associated with this gene variation and homocysteine levels.

**Table 1:** Pharmacologic substrates, inhibitors and inducers of cytochrome P450 (CYP2D6) of relevant psychotropic drugs.

Substrate	Inhibitors	Inducers
**Acetaminophen**	Amiodarone	Dexamethasone
**Amphetamine-Dextroamphetamine**	Bupropion	Rifampin
**Aripiprazole**	Celecoxib	
**Atomoxetine**	Chlorpheniramine	
**Clonidine**	Chlorpromazine	
Codeine *	**Citalopram**	
**Dextromethorphan**	Clozapine	
Duloxetine	Cocaine	
**Fluoxetine**	Desipramine	
**Fluvoxamine**	**Diphenhydramine**	
Haloperidol	Duloxetine	
Iloperidone	**Fluoxetine**	
Methadone	Halofantrine	
Methamphetamine	Haloperidol	
Mirtazapine	Hydroxychloroquine	
Nefazodone	Imatinib	
Olanzapine	**Imipramine**	
Paroxetine	Levomepromazine	
Phenothiazines	Methadone	
Risperidone *	Metoclopramide	
**Sertraline**	Mibefradil	
**Tricyclic antidepressants (TCAs)**	Moclobemide	
Tramadol	Nelfinavir	
Venlafaxine *	Norfluoxetine	
Vortioxetine	Paroxetine	
	Perphenazine	
	Quinidine	
	Ranitidine	
	Ritonavir	
	**Sertraline**	
	Terbinafine	
	Thioridazine	
	Tranylcypromine	

Phenothiazines include: Chlorpromazine, fluphenazine, perphenazine, promethazine, thioridazine; tricyclic antidepressants include: Amitriptyline, amoxapine, clomipramine, desipramine, doxepin, imipramine, nortriptyline, protriptyline, trimipramine; * Metabolized to active compound; ω-3 fatty acids can inhibit CYP2D6 activity at high doses [28]; Compounds prescribed for our clinical case are indicated in **bold**. Table revised from literature supplied by Genomind, LLC (www.genomind.com).

Additionally, our clinical case had CYP2D6*4/*5 gene allele variation that indicated significant reduction in enzyme activity. The *4 variation represents a G to A transition at the first nucleotide of exon 4 of one allele while the *5 variation represents a deletion of the second allele [29]. This is likely to put the patient at risk for significantly reduced hepatic degradation of targeted drugs and higher plasma levels of drugs that are typically processed by this enzyme thereby increasing the risk for drug interactions and reduced effectiveness of medications such as risperidone [13,30,31,32,33,34,35,36,37,38,39,40,41,42,43,44,45,46,47,48,49,50,51,52,53]. Caution should be used when prescribing medications that require this enzyme for metabolic break down. It would be important to avoid prescribing any inhibitors of CYP2D6, as well, which includes other medications that may lower further the enzymatic activity. On the other hand, inducers of CYP2D6 would increase the metabolic activity of CYP2D6. Table 1 lists psychotropic drugs known to be processed by CYP2D6, as well as inhibitors and inducers of this enzyme activity [54,55].

Many drugs prescribed for our patient (aripiprazole, dextroamphetamine, fluoxetine, fluvoxamine) were dependent on normal CYP2D6 enzyme activity and metabolism for degradation while other drugs such as risperidone require conversion to a therapeutic agent using this enzyme. Disruption of CYP2D6 function may partially or completely explain problems experienced by our clinical case when using these drugs. Some of the medications used were also inhibitors of CYP2D6 (e.g., citalopram, sertraline) which are expected to further suppress the reduced activity (see Table 2). Also, dextomethoraphan (DM) is an ingredient found in Nyquil Cold Medicine and used as a cough suppressant. It is a non-psychotropic medication substrate of CYP2D6 and excreted by the kidneys. It has a half-life of 2 to 4 h (for those with extensive (normal) metabolism) but 24 h for those individuals categorized as poor metabolizers (as found in our clinical case). Directions for use of Nyquil Cold Medication are not to exceed 4 doses in 24 h and with this recommended dosage, the amount of DM would be expected to be increased in the blood stream in those with poor metabolism. DM acts as an NMDA receptor antagonist at high doses and produces dissociative states similar to what is seen by other dissociative anesthetics such as ketamine and phencyclidine. When exceeding label-specified maximum dosages, dextromethorphan can thus act as a dissociative hallucinogen including visual field disturbances, distorted bodily perception and excitement.

**Table 2:** Pharmacogenetic test results with drug interactions from our clinical case.

Pharmacogenetic Target	Variant Functional Impact	Compounds Prescribed
**CYP2D6**	Poor cytochrome p450 metabolism	Acetaminophen, Aripiprazole, Atomoxetine, Citalopram, Dextroamphetamine, Dextromethorphan, Fluoxetine, Fluvoxamine, Risperidone *, Sertraline
**5HT2C**	Reduced affinity for serotonin	Fluoxetine, Fluvoamine, Sertraline
**MTHFR**	Reduced activity (low monoamine and catecholamine production)	Methyl/folate-related agents (vitamins)

* Metabolized to active compound.

As a slow or poor metabolizer, our clinical case would likely experience an increased prolonged sedative effect which was noted by his parents during the time he was using Nyquil Cold Medication [56,57]. Of interest, escitalopram is not dependent on CYP2D6 for metabolism but is dependent on both CYP3A4 and CYP2C19 [53], different p450 enzymes which were found to be normal by gene polymorphism testing in our clinical case. Ultimately, escitalopram, which was prescribed, was found to be the most effective medication to date for treating his behavioral problems and supported by his pharmacogenetic testing results.

## 3. Experimental Section

Saliva was collected from our patient in June 2014 when he was 16 years of age. DNA was isolated from saliva samples and sent for pharmacogenetics Genecept DNA-based assay commercially available from Genomind (Chalfont, PA, USA). The Genecept assay examines polymorphisms of selected genes for CYP450 hepatic enzymes which metabolize drugs as well as other genes related to neurotransmitters, their function, receptors and enzymes implicated in psychiatric disorders, and responsiveness to psychiatric medications. The list includes ten separate genes encoding cytochrome p450 enzymes with three related to the metabolism of pharmaceutical agents commonly used in psychiatry (*CYP2D6*, *CYP2C19*, *CYP3A4*) and seven additional genes encoding neurotransmitter receptors (*5HT2C*, *DRD2*), transporters (*SLC6A4*), enzymes (*COMT*, *MTHFR*) and ion channel function (*CACNA1C*, *ANK3*) involved in pharmacodynamic aspects of psychiatric medications. 

## 4. Conclusions

It is clear in this clinical case that certain recommendations for care could be made due to pharmacogenetic findings. An approach would be to select a different class or drug that has a similar function but metabolized by a different CYP450 enzyme. The dosage or frequency of drug of administration of the specific medication could be adjusted to account for the metabolic disturbance (poor or ultrametabolizer). One should also consider other prescribed drugs or over the counter that may induce or inhibit the specific CYP450 enzyme (Table 1 shows a list of medications/agents that either induce or inhibit CYP2D6 activity). For example, eicosapentaenoic acid (EPA) is an ω-3 fatty acid and acts as an inhibitor of CYP2D6 [28] at higher doses as are antihistamines such as diphenhydramine [58]. Dietary considerations, such as the addition of folic acid or methyl group donor compound can decrease homocysteine blood levels and thus lower the negative impact from the *MTHFR* gene variant seen in our clinical case.

In summary, the history of our clinical case illustrates how pharmacogenetics and psychiatry can potentially interface to provide more informed decision making regarding use of psychotropic medications. Pharmacogenetics testing is new to the field of psychiatry and in similar situations may appear to be more humane and likely save years of personal distress associated with trial and error drug prescription without taking into consideration an individual’s specific drug metabolism pattern. It would also likely be more cost effective to prescribe psychotropic medications with the more informed decision making provided by this type of genetic testing. The cost of testing (approximately $400) is low when compared to the financial costs of many other treatments utilized by this clinical case over several years. The current report involving only one clinical case is limited in generalizability and more clinical reports are needed to help guide treatment for providers. However, if similar experiences and clinical case studies are found then pharmacogenetic testing should become standard of care in mental health treatment and decision making when using psychotropic medications. Sharing these experiences would be encouraged by the authors.
